# MXene-Based Electrochemical Biosensors: Advancing Detection Strategies for Biosensing (2020–2024)

**DOI:** 10.3390/bios15030127

**Published:** 2025-02-20

**Authors:** Joydip Sengupta, Chaudhery Mustansar Hussain

**Affiliations:** 1 Department of Electronic Science, Jogesh Chandra Chaudhuri College, Kolkata 700033, India; joydipdhruba@gmail.com; 2 Department of Chemistry and Environmental Science, New Jersey Institute of Technology, Newark, NJ 07102, USA

**Keywords:** MXenes, electrochemical biosensors, biomedical applications, cancer biomarker, pathogens, bodily metabolites, chemical messenger

## Abstract

MXenes, a class of two-dimensional materials, have emerged as promising candidates for developing advanced electrochemical biosensors due to their exceptional electrical conductivity, large surface area, and rich surface chemistry. These unique properties enable high sensitivity, rapid response, and versatile functionalization, making MXene-based biosensors highly suitable for detecting biomolecules and pathogens in biomedical applications. This review explores recent advancements in MXene-based electrochemical biosensors from 2020 to 2024, focusing on their design principles, fabrication strategies, and integration with microfluidic platforms for enhanced performance. The potential of MXene sensors to achieve real-time and multiplexed detection is highlighted, alongside the associated challenges. Emphasis is placed on the role of MXenes in addressing critical needs in disease diagnostics, personalized medicine, and point-of-care testing, providing insights into future trends and transformative possibilities in the field of biomedical sensing technologies.

## 1. Introduction

The demand for advanced diagnostic technologies has surged in response to the increasing complexity of biomedical challenges, including early disease detection, precision medicine, and real-time health monitoring. Electrochemical biosensors [1] have emerged as a transformative tool in this landscape, offering unparalleled advantages such as high sensitivity, selectivity, rapid response, portability, and scalability. By converting biochemical interactions into measurable electrical signals, these devices provide a robust platform for detecting a broad spectrum of biological analytes [2]. The continued evolution of biosensor technologies, however, requires the development of innovative materials that enhance performance and expand their applicability across diverse biomedical fields.

**Figure 1:**
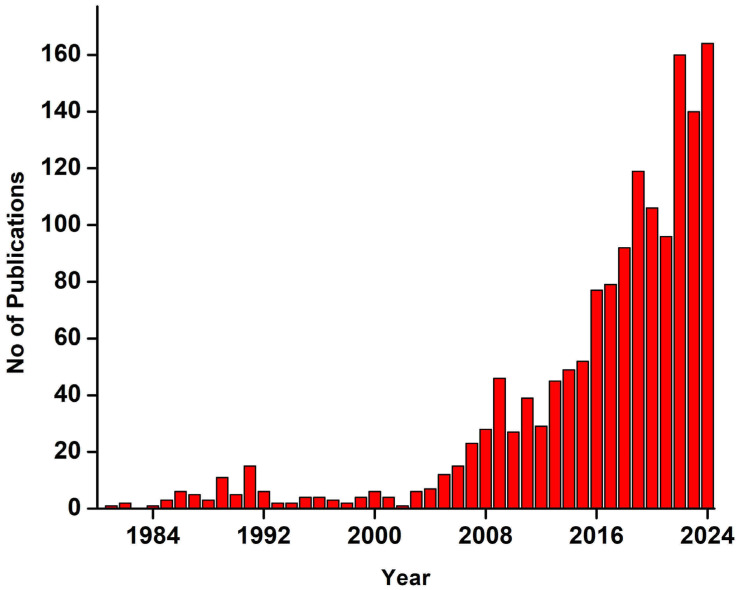
Trend of research publications on electrochemical biosensors for biomedical applications, retrieved from Scopus (Elsevier) using the search term “TITLE-ABS-KEY (biosensor AND electrochemical AND biomedical)”.

The graph (Figure 1) illustrates the growing research interest in electrochemical biosensors for biomedical applications, based on Scopus-indexed publications. The exponential increase in publications, particularly after 2010, indicates the expanding relevance of this field in healthcare diagnostics, disease monitoring, and personalized medicine. The sharp rise in recent years underscores the advancements in sensor technology, miniaturization, and interdisciplinary integration with nanotechnology. This trend reflects the critical role of electrochemical biosensors in addressing global healthcare challenges through rapid, cost-effective, and highly sensitive diagnostic tools.

Electrochemical biosensors hold significant promise for revolutionizing point-of-care (POC) diagnostics due to their inherent advantages of portability, rapid response, and cost-effectiveness [3]. These devices integrate biological recognition elements with electrochemical transducers, enabling real-time or near-real-time detection of target analytes in complex biological samples. Their miniaturization potential allows for the development of compact, handheld systems that can be deployed in resource-limited settings, remote areas, or even at home, bypassing the need for centralized laboratory infrastructure. For instance, electrochemical biosensors have demonstrated utility in POC applications such as glucose monitoring for diabetes management [4], rapid detection of infectious diseases (e.g., COVID-19 antigen tests) [5], and screening for cardiac biomarkers during emergencies [6]. Their ability to operate with minimal sample volumes and provide quantitative results with high sensitivity and specificity aligns with the growing demand for decentralized healthcare solutions. Furthermore, advancements in nanomaterials, flexible electronics, and wireless connectivity are enhancing their multiplexing capabilities and integration with digital health platforms, enabling seamless data transmission to healthcare providers. By bridging the gap between laboratory-grade accuracy and on-site usability, electrochemical biosensors are poised to improve early diagnosis, personalized treatment, and real-time health monitoring, ultimately transforming global healthcare accessibility and outcomes.

Electrochemical biosensors have emerged as powerful tools in modern healthcare, offering rapid, cost-effective, and portable solutions for detecting biomarkers, pathogens, and metabolites. However, their widespread clinical adoption is hindered by several limitations [7], including challenges in selectivity within complex biological matrices like blood or saliva, where interferents such as proteins and lipids can generate false signals, compromising diagnostic accuracy for conditions like sepsis or cancer. Sensitivity limitations further restrict their ability to detect ultralow analyte concentrations, delaying timely intervention [8]. Stability and reproducibility issues arise from the degradation of biorecognition elements (e.g., enzymes, antibodies) and batch-to-batch fabrication inconsistencies, eroding clinician trust and complicating regulatory approval [9]. Miniaturization and integration challenges also persist, as balancing portability with analytical performance remains difficult, limiting the utility of wearable or POC devices [10]. Cost barriers, particularly for sensors relying on precious metals or complex manufacturing, further hinder scalability and accessibility in resource-limited regions. Clinically, these limitations affect both established applications, such as glucose monitoring, which still faces biofouling and recalibration issues [11], and emerging applications, such as neurotransmitter [12] or cardiac biomarker detection [13], which struggle to match the sensitivity and specificity of gold-standard lab techniques. Addressing these challenges requires multidisciplinary innovation, including advances in nanomaterials.

Among the various materials investigated, MXenes—a novel class of two-dimensional (2D) transition metal carbides, nitrides, and carbonitrides—have garnered significant interest due to their extraordinary physicochemical properties [14]. First discovered in 2011 [15], MXenes have since emerged as versatile materials, combining metallic conductivity, hydrophilicity, high surface-to-volume ratio, and ease of functionalization. These unique characteristics make them highly attractive for biosensing applications, where they can serve as signal transduction layers [16], immobilization platforms [17], or hybrid materials in conjunction with other nanomaterials [18]. Their layered structure also provides abundant active sites for functionalization with biomolecules, enabling the development of highly selective and sensitive biosensors tailored for specific analytes. However, other 2D nanomaterials, such as graphene, transition metal dichalcogenides (TMDs), and hexagonal boron nitride (h-BN), also play critical roles in sensing technologies. Each of these materials possesses distinct characteristics that make them suitable for specific applications.

The following table (Table 1) provides a comparative overview of MXene with other prominent 2D nanomaterials.

In the realm of biomedical diagnostics, MXene-based electrochemical biosensors have demonstrated exceptional potential in detecting clinically relevant biomarkers, such as proteins [19], RNA [20], small molecules [21], and pathogens [22]. Their ability to achieve ultra-low detection limits (LODs) [23] and high signal stability has positioned them as key players in addressing the challenges of early disease detection and therapeutic monitoring. Furthermore, their compatibility with wearable and implantable electronics has opened new avenues for real-time, POC diagnostics, aligning with the growing emphasis on personalized healthcare solutions [22].

This review aims to provide an in-depth exploration of the rapidly expanding field of MXene-based electrochemical biosensors from 2020 to 2024. Additionally, it highlights recent advances in MXene-enabled detection strategies for various biomedical applications, emphasizing their role in transforming diagnostic methodologies. By synthesizing the current state of knowledge and identifying future research directions, this review seeks to inspire further innovations in the development of MXene-based biosensors, ultimately contributing to the advancement of biomedical science and healthcare technologies.

## 2. MXene: Structure, Synthesis and Properties

### 2.1. MXene: Structure

**Figure 2:**
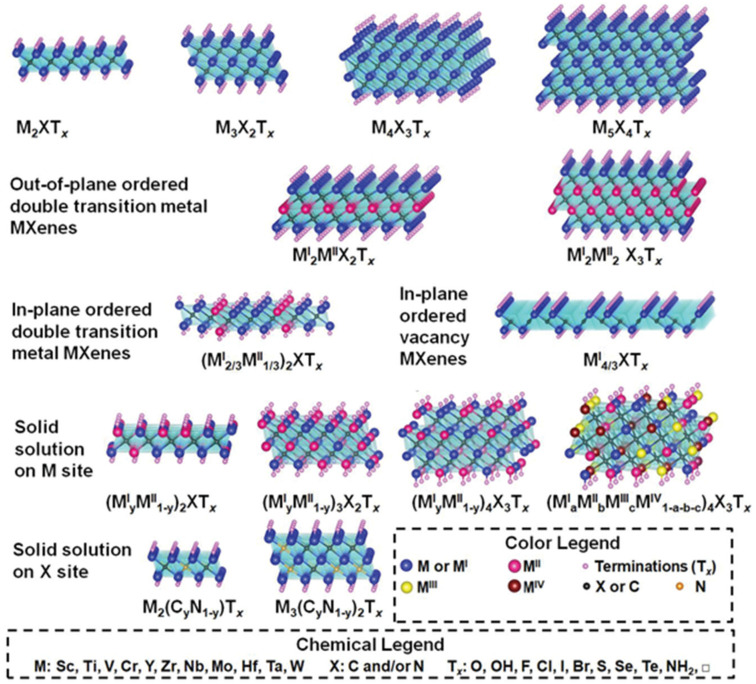
Representative structures and compositions of MXenes. MXenes are characterized by the general formula M_n+1_X_n_T_x_, where M denotes an early transition metal, X represents carbon (C) or nitrogen (N), and T_x_ indicates surface terminations such as -OH, -O, or -F. The n value, which determines the structural composition, can range from 1 to 4. The M-sites may be occupied by one, two, or more transition metals, leading to the formation of solid solutions or distinct ordered structures. Notably, double-transition-metal MXenes can exhibit unique arrangements, including in-plane ordered structures (i-MXenes), in-plane vacancy configurations, and out-of-plane ordered structures (o-MXenes), where one or two layers of a secondary transition metal M^II^ are intercalated between layers of the primary transition metal M^I^ (reproduced with permission from [28]).

MXenes are a class of 2D materials derived from the selective etching of the A-layer in the parent MAX phases [24], which are ternary carbides or nitrides with the formula M_n+1_AX_n_. In these phases, M is an early transition metal, A is typically a group 13 or 14 element, and X represents carbon or nitrogen. The resulting MXenes have the general formula M_n+1_X_n_T_x_, where T_x_ refers to surface terminations, such as -OH, -O, or -F. These terminations enhance the hydrophilicity of the material and provide avenues for further chemical modification, giving MXenes a high degree of versatility [25]. The layered structure of MXenes consists of metal atoms arranged in a hexagonal lattice, interspersed with X-atoms, and bound by weaker van der Waals forces. The number of layers, denoted by *n*, can vary from one to four, with this structural flexibility impacting the properties of the material, such as its conductivity and mechanical strength [26]. MXenes also exhibit structural diversity in M-site configurations. Single-transition-metal MXenes have identical M-atoms, while double-transition-metal MXenes incorporate two distinct metals, forming ordered structures (i-MXenes and o-MXenes) [27]. Additionally, in-plane vacancy structures introduce tunable defects affecting their properties (Figure 2) [28].

### 2.2. MXene: Synthesis

The synthesis of MXenes involves the selective removal of the A-layer elements from MAX phases, which are ternary carbides or nitrides with the general formula M_n+1_X_n_T_x_ [29].

The preparation of MAX phase precursors begins with powder mixing, where metal powders, carbon or nitrogen sources, and the A-element are combined in precise stoichiometric ratios [30]. This mixture is then compacted into pellets and subjected to inert sintering at temperatures ranging from 1200 to 1600 °C in an argon atmosphere to promote diffusion while preventing oxidation [31]. The sintering process facilitates the formation of the desired MAX phase structure, which is verified using various characterization techniques after cooling to ensure the proper phase for subsequent MXene production [32].

**Figure 3:**
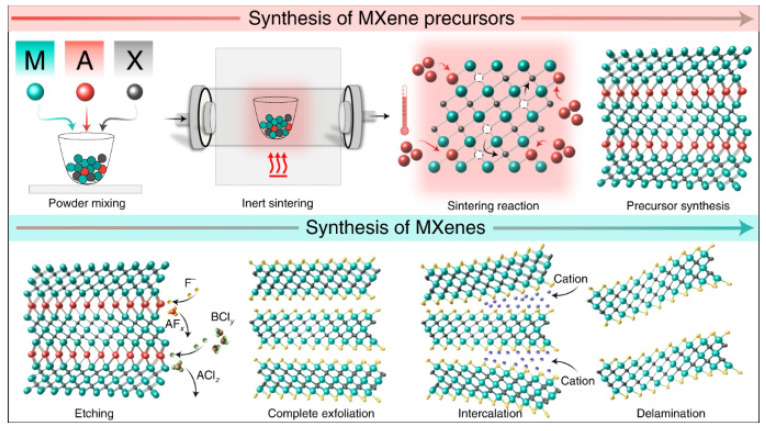
Synthesis of MXenes (reproduced with permission from [38]).

The synthesis of MXenes starts with etching, wherein the A-element (e.g., Al) is selectively removed from the MAX phase using strong acids such as hydrofluoric acid (HF) [33] or alternative fluoride-free methods [34]. This etching step results in a layered structure composed of transition metals and carbon or nitrogen, with surface terminations (e.g., -OH, -O, -F) introduced during the process. Following etching, exfoliation is performed to separate the individual MXene layers. This is commonly achieved by using intercalating agents like dimethyl sulfoxide (DMSO), which penetrate the interlayer spaces and weaken the van der Waals forces holding the layers together [35]. Intercalation further enhances exfoliation by inserting ions or molecules between the MXene layers, which can modify the interlayer spacing and surface chemistry of the material [36]. Finally, delamination is carried out through mechanical agitation or sonication [37], fully separating the individual MXene sheets to produce high-quality, single- or few-layer MXenes (Figure 3).

### 2.3. MXene: Properties

The properties of MXenes are governed by their intrinsic atomic structure, surface functionalization, and interlayer spacing adjustments introduced during synthesis [39]. MXenes exhibit exceptional electrical conductivity (up to 20,000 S/cm) and tunable work function (1.6–6.25 eV) [40], attributable to the metallic bonding of transition metal carbides/nitrides and electron-rich surface terminations [41]. Mechanically, they demonstrate remarkable flexibility combined with high tensile strength (15.4 GPa) and Young’s modulus (0.484 ± 0.013 TPa) for 2D Ti_3_C_2_T_x_ MXene monolayers [42], enabling their integration into deformable electronics and wearable biosensors [43].

Surface functionalization plays a crucial role in enhancing the performance of MXene-based biosensors by improving sensitivity, stability, and selectivity. Enzyme immobilization on MXene surfaces, achieved through covalent bonding or physical adsorption, enhances catalytic activity and facilitates rapid electron transfer, making them ideal for electrochemical sensing applications [44]. Aptamer binding, utilizing thiol–gold interactions or π–π stacking with MXene’s 2D structure, ensures high specificity for target analytes such as pathogens and biomarkers, allowing for precise and reusable detection [45]. Polymer coatings further optimize sensor performance by reducing biofouling, enhancing biocompatibility, and improving electron conductivity, with conductive polymers like polypyrrole facilitating efficient charge transfer [46]. Surface functionalization plays a critical role in tailoring MXene properties. The most common terminations (–O, –OH, –F), inherited from synthesis etchants like HF or LiF/HCl, modulate hydrophilicity, chemical reactivity, and interlayer spacing [47]. The large specific surface area (200.8 m^2^/g) [48] and tunable surface chemistry facilitate high-density biomolecule immobilization, a key feature for biosensing.

Biocompatibility is another pivotal property for biomedical applications [49]. In vitro and in vivo studies confirm that MXenes exhibit low cytotoxicity at concentrations below 100 μg/mL, particularly when –OH terminations dominate, as they minimize reactive oxygen species (ROS) generation [50]. However, biocompatibility is concentration- and termination-dependent [51]: excessive –F groups or prolonged exposure to unshielded MXenes can induce cellular stress, necessitating surface passivation strategies (e.g., polymer coatings) for therapeutic devices [52].

Despite these advantages, environmental susceptibility remains a challenge. MXenes oxidize in humid or aqueous environments. Recent advances in covalent functionalization (e.g., silane grafting) [53] has improved stability, though long-term performance under physiological conditions requires further optimization [54].

## 3. Electrochemical Biosensors and Integration of MXenes

### 3.1. Electrochemical Biosensor Architecture

**Scheme 1:**
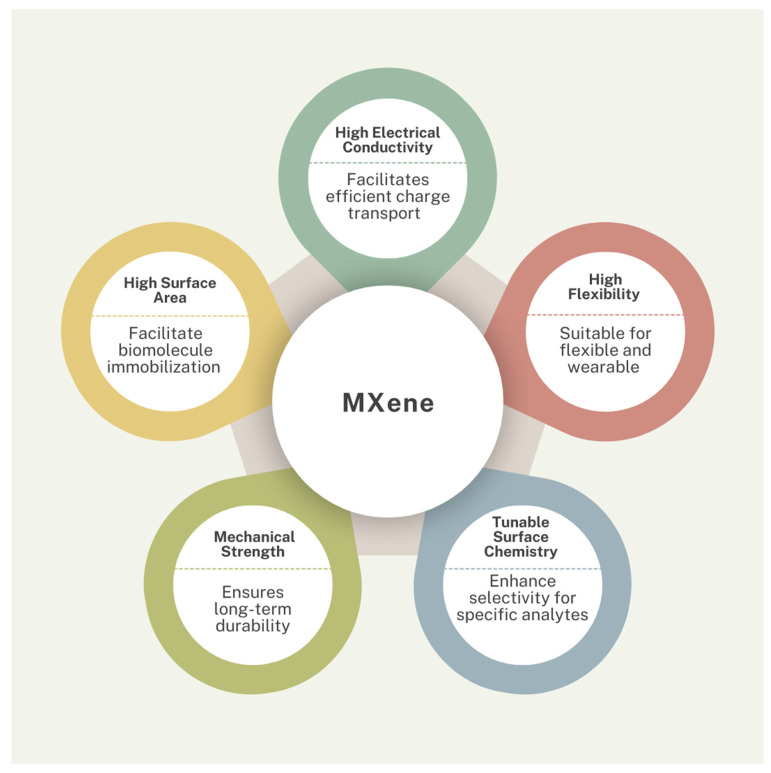
Key attributes highlighting the integration potential of MXene in biosensor applications.

Electrochemical biosensors [55] are crucial for detecting biological markers, pathogens, and analytes due to their high sensitivity, rapid response, and portability [56]. A typical biosensor consists of a bioreceptor (e.g., enzymes, antibodies, or DNA) for selective analyte recognition, a transducer that converts biochemical interactions into measurable electrical signals, and an electrode system (working, reference, and counter electrodes) that governs signal acquisition [57]. The electrode material plays a critical role in determining performance by influencing sensitivity, stability, and signal-to-noise ratios [58]. MXenes, characterized by their high surface area and tunable surface chemistry, have demonstrated exceptional potential as electrode materials (Scheme 1), facilitating efficient bioreceptor immobilization and enhanced electron transfer [59].

### 3.2. Transduction Mechanisms

Electrochemical biosensors operate through transduction mechanisms that correlate biochemical events with electrical outputs [60]. Amperometric detection measures current changes resulting from redox reactions at the electrode surface [61], where MXenes’ high electrical conductivity enhances electron transfer, leading to faster response times and lower detection limits [62]. Potentiometric detection monitors potential differences that arise from ion-selective interactions [63], and MXenes’ surface terminations (e.g., –OH, –F) allow for precise ion adsorption tuning, thereby improving selectivity. Impedimetric detection tracks impedance variations during analyte binding, benefiting from MXenes’ large surface area, which amplifies signal sensitivity by increasing biomolecule–electrode interactions [64]. The synergistic properties of MXenes optimize these transduction mechanisms, enhancing signal amplification while reducing nonspecific binding [65].

MXenes play a crucial role in improving electrochemical transduction mechanisms by enhancing both signal amplification and charge transfer efficiency. In signal amplification, MXenes act as excellent platforms for immobilizing enzymes, aptamers, and nanocatalysts, significantly enhancing electrochemical responses [23]. Their large surface area promotes a higher loading capacity for biorecognition elements, leading to increased target binding events and amplified signal outputs. Additionally, the synergistic integration of MXenes with other nanomaterials, such as metal nanoparticles and carbon-based materials, further boosts electrocatalytic activity, improving detection limits and response times [66].

Regarding charge transfer, MXenes exhibit metallic or semiconducting behaviour depending on their composition and functionalization, enabling efficient electron transport between the sensing interface and the electrode [67]. Their ultra-thin structure reduces charge transfer resistance, facilitating faster redox reactions and improved current responses. Moreover, MXenes’ hydrophilic nature [68] ensures uniform dispersion in biosensor matrices, enhancing electrode–solution interactions and overall sensor performance. These properties collectively contribute to the development of highly sensitive, selective, and rapid electrochemical biosensors for biomedical and environmental applications.

### 3.3. Assembly and Elaboration Strategies for MXene Incorporation

The incorporation of MXenes into biosensor architectures involves strategic assembly techniques to maximize their functional advantages [2]. Direct electrode modification is commonly achieved by drop-casting or spin-coating MXenes onto electrode surfaces [69], forming conductive thin films with uniform coverage, even on flexible substrates suitable for wearable biosensors. Biomolecule functionalization [70] utilizes MXenes’ surface chemistry for covalent or non-covalent bioreceptor immobilization (e.g., antibodies via carbodiimide coupling), ensuring stability while preserving bioreceptor activity. Composite fabrication, which combines MXenes with graphene [71], noble metal nanoparticles [72], or polymers, has been employed to enhance catalytic activity and prevent MXene aggregation, with MXene-AuNP composites demonstrating amplified redox signals in amperometric sensors. Additionally, layer-by-layer (LBL) assembly alternates MXene layers with bioreceptors or polymers, generating hierarchically structured interfaces that optimize analyte capture and signal transduction [73].

### 3.4. Advanced Elaboration Techniques

Emerging strategies aim to refine MXene interfaces for specific biosensing applications. Pore engineering techniques [74] involve the fabrication of MXene aerogels or mesoporous films to increase the number of active sites available for biomolecule loading, thereby improving sensor sensitivity. Surface passivation through biocompatible polymer coatings, such as chitosan, has been implemented to mitigate fouling in complex biological samples, extending biosensor longevity [75]. The integration of MXene-based electrodes into microfluidic systems [76] facilitates automated, high-throughput analyte detection, further enhancing biosensor performance for real-time diagnostics.

MXenes’ unique combination of high conductivity, tunable surface chemistry, and mechanical robustness [77] has significantly advanced electrochemical biosensor design. By optimizing biosensor architectures, transduction mechanisms, and assembly strategies, MXene-enhanced biosensors have demonstrated remarkable improvements in sensitivity, stability, and versatility. These advancements position MXene-based biosensors as powerful tools for POC diagnostics and environmental monitoring, paving the way for next-generation sensing technologies [78].

## 4. Biomedical Applications of MXene-Based Electrochemical Biosensors

### 4.1. Applications of MXene-Based Electrochemical Biosensors in Detecting Cancer Biomarkers

MXene-based electrochemical biosensors have emerged as a promising platform for the sensitive and reliable detection of cancer biomarkers, offering significant advancements in early diagnostic techniques. These biosensors, leveraging the unique properties of MXene nanomaterials, demonstrate remarkable performance in detecting a variety of cancer-related biomarkers, facilitating more efficient monitoring and targeted therapeutic strategies.

**Figure 4:**
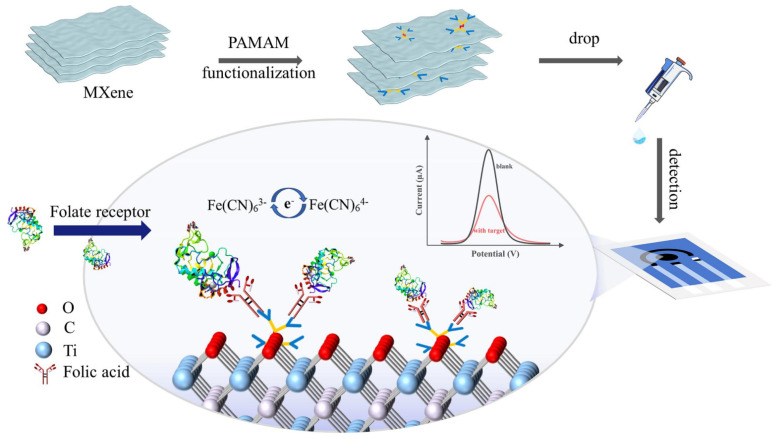
A schematic illustration of the PAMAM@MXene synthesis process and the sensing mechanism for FR detection (reproduced with permission from [79]).

Folate receptor (FR) expression was widely recognized as a critical biomarker for assessing tumour-associated pathologies, with its accurate quantification regarded as essential for early diagnostic efforts and the development of targeted therapeutic strategies. Advancing electrochemical biosensors required a focus on optimizing electrode modifications to enhance sensitivity and detection performance. A highly conductive and stable nanocomposite was successfully synthesized by Jiang et al. [79] via in situ grafting first-generation poly(amidoamine) (PAMAM) dendrimers onto MXene (Ti_3_C_2_T_x_), resulting in a robust immobilization matrix designated as PAMAM@MXene. This nanocomposite was utilized to construct an electrochemical sensor by integrating PAMAM@MXene onto screen-printed carbon electrodes (SPCEs) for FR detection (Figure 4). The sensor exhibited excellent performance under optimized conditions, achieving reliable quantification of FR over a broad concentration range of 10–1000 ng/mL with an LOD of 5.6 ng/mL. Additionally, the sensor demonstrated high selectivity, reproducibility, and stability, underscoring its significant potential for clinical applications in FR monitoring and advancing tumour diagnostic technologies.

The clinical significance of the ratio of free prostate-specific antigen (fPSA) to total prostate-specific antigen (tPSA) within the “grey zone” of tPSA levels, ranging from 4 ng/mL to 10 ng/mL, was well established in the diagnosis of prostate cancer. However, the precise quantification of this ratio remained challenging due to the extremely low concentrations of fPSA and tPSA in peripheral blood and the high complexity of biological samples. To address these limitations, Zhu et al. [46] developed a biosensor based on interdigitated spiral MXene-assisted organic electrochemical transistors (isMOECTs) for the sensitive detection of the fPSA/tPSA ratio. The integration of MXene (Ti_3_C_2_) nanomaterials with an innovative interdigitated multiple spiral architecture synergistically amplified the amperometric signals through dual functionalization with anti-tPSA and anti-fPSA antibodies. The biosensor’s ultrasensitivity was achieved through the tunable spiral structure and the exceptional properties of MXene, enabling an LOD of 0.01 pg/mL for both tPSA and fPSA. Furthermore, the sensor demonstrated excellent selectivity, reproducibility, and stability, highlighting its robustness for practical applications. These findings underscored the reliability and efficacy of MXene-assisted organic electrochemical transistor biosensors with interdigitated spiral designs, offering promising opportunities for POC diagnostic applications in clinical settings.

The detection of carcinoembryonic antigen (CEA) is crucial for the early diagnosis and management of colorectal cancer (CRC), as it aids in identifying cancer at stages when treatment is most effective. For the efficient detection of CEA, an innovative electrochemical biosensing platform was developed by Kalkal et al. [71] using an f-graphene@Ti_3_C_2_-MXene nanohybrid thin film. The platform was fabricated by employing an air-brush spray coating technique to deposit uniform thin films of amine-functionalized graphene (f-graphene) and Ti_3_C_2_-MXene nanohybrid onto ITO-coated glass substrates. To facilitate the specific binding of CEA, the deposited films were functionalized with monoclonal anti-CEA antibodies via EDC-NHS chemistry, followed by the blocking of nonspecific binding sites with bovine serum albumin (BSA). The electrochemical performance of the biosensor was evaluated and optimized through cyclic voltammetry (CV) and differential pulse voltammetry (DPV). The resulting immunoelectrode, comprising BSA/anti-CEA/f-graphene@Ti_3_C_2_-MXene, exhibited a wide detection range for CEA, from 0.01 pg/mL to 2000 ng/mL, with a notable LOD of 0.30 pg/mL, demonstrating the platform’s high sensitivity and potential for CEA biomarker detection.

**Figure 5:**
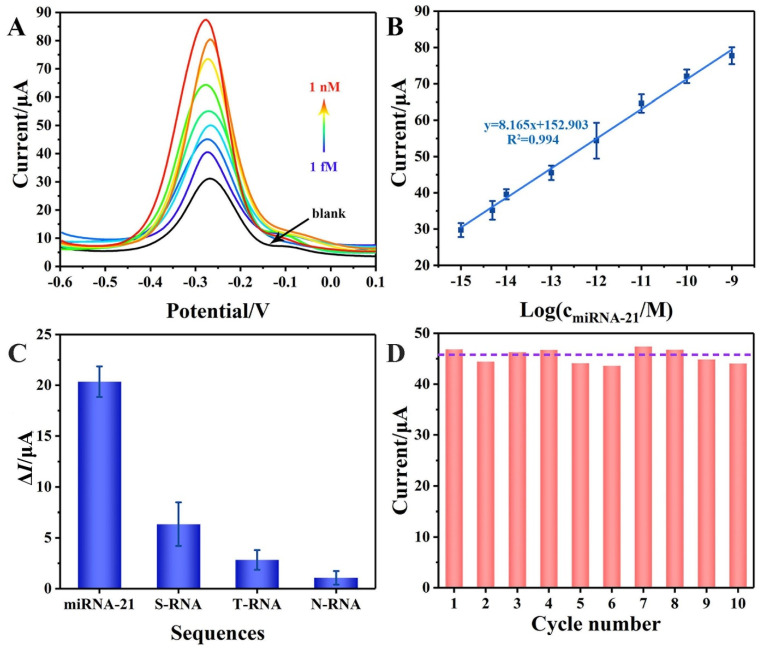
(**A**) The DPV curves of the MXene-rGO-Au-based biosensor were recorded for miRNA-21 solutions with concentrations of 0, 1, 5, 10, and 100 fM, as well as 1, 10, 100, and 1 nM, respectively. (**B**) The DPV current responses were plotted against the logarithmic concentrations of miRNA-21 for the developed biosensor. (**C**) The effect of hybridization with different 100 fM miRNA-21 concentrations on the performance of the MXene-rGO-Au-based biosensor was assessed. (**D**) The peak currents of the MXene-rGO-Au-based biosensor were measured across ten repeated measurements at 100 fM concentration (reproduced with permission from [20]).

The detection of microRNAs has been recognized as a key tool in the early identification of diseases such as cancer. An advanced, label-free biosensor was developed by Guo et al. [20] to enable the rapid and ultrasensitive detection of miRNA-21. The biosensor integrated MXene-reduced graphene oxide (rGO)–gold (Au) as the electrode material for efficient DNA probe immobilization, with MXene-AuPd nanocomposites incorporated to facilitate signal amplification. This biosensor exhibited an extensive linear detection range, from 1 fM to 1 nM, and achieved an extraordinary LOD of 0.42 fM under optimized conditions (Figure 5). Moreover, the device demonstrated exceptional specificity, stability, and reproducibility, making it capable of accurately detecting miRNA-21 in real samples from both murine and human sources, with remarkable sensitivity.

Breast cancer, recognized as the second most prevalent malignancy globally, remains a major contributor to cancer-related mortality worldwide. In response to this challenge, a novel electrochemical biosensor was developed by Ranjbari et al. [23] via the integration of hierarchical flower-like gold, poly(n-butyl acrylate), and MXene (AuHFGNs/PnBA-MXene) nanocomposite, which was activated by a highly specific antisense single-stranded DNA (ssDNA). This biosensor was fabricated to detect miRNA-122, a biomarker linked to breast cancer. The sensor demonstrated a remarkable LOD of 0.0035 aM and exhibited a broad linear detection range from 0.01 aM to 10 nM. Its performance was evaluated using 20 serum samples from breast cancer patients, including 10 positive and 10 negative cases. Statistical methods such as *t*-tests, box plots, receiver operating characteristic (ROC) curves, and cutoff analysis were applied to the quantitative results. The biosensor showed perfect specificity and sensitivity (100%) in differentiating between positive and negative samples. Furthermore, the ssDNA/AuHFGN/PnBA-MXene/GCE sensor demonstrated minimal cross-reactivity with other sequences and exhibited excellent stability, reproducibility, and specificity, maintaining its performance over 32 days.

The detection of miRNA-155 is crucial as it serves as a promising noninvasive biomarker for early diagnosis of various cancers. A highly sensitive electrochemical biosensor for miRNA-155 detection was constructed by Yang et al. [72], employing a 3D nanocomposite of gold nanoparticles (AuNPs) and Ti_3_C_2_ MXene, combined with exonuclease III (Exo III)-mediated cascade target recycling. The Ti_3_C_2_ MXene, synthesized for its distinctive layered structure and metallic properties, provided a robust platform for the immobilization of AuNPs. The AuNPs enabled the covalent immobilization of capture DNA (C-DNA) functionalized with methylene blue (MB) at the 3′ terminus, which produced an initial electrochemical signal measured using DPV. Upon exposure to miRNA-155, hybridization with C-DNA formed a double-stranded structure that activated Exo III-mediated cleavage at the 3′ end of the C-DNA, leading to a reduction in the electrochemical signal. This Exo III-driven cascade cleavage mechanism released multiple C-DNA strands, significantly amplifying the signal difference. The biosensor achieved an exceptional LOD of 0.35 fM and exhibited a broad linear detection range from 1.0 fM to 10 nM. Moreover, it demonstrated excellent stability, reproducibility, and specificity, highlighting its potential for the accurate and reliable detection of miRNA-155 in complex biological samples.

### 4.2. Applications of MXene-Based Electrochemical Biosensors in Detecting Pathogens

**Figure 6:**
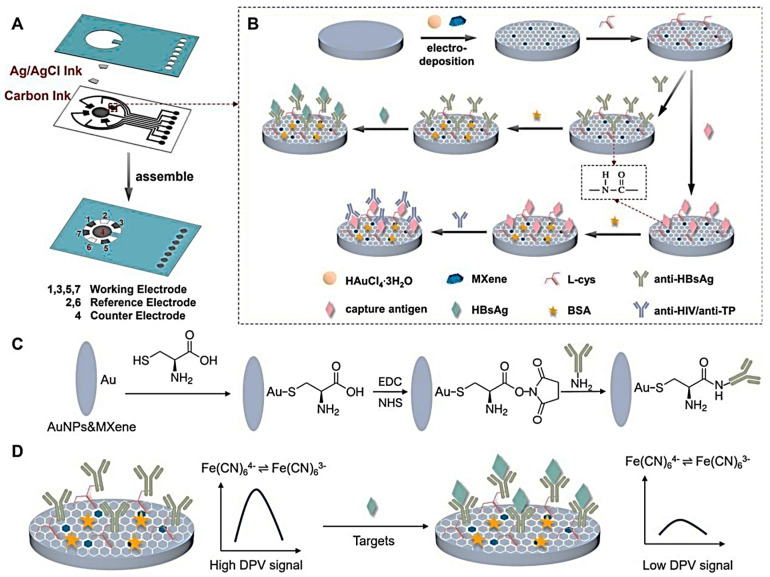
A schematic representation of the fabrication process and detection mechanism of the AuNPs&MXene-SPE sensor is presented. (**A**) The architectural layout and sequential development of the screen-printed electrodes are detailed. (**B**) The step-by-step fabrication of the AuNPs&MXene-SPE sensor is systematically carried out on the working electrodes. (**C**) The electrode functionality is achieved by labelling L-cysteine (L-Cys) on the electrode surface through the formation of Au-S bonds, followed by the immobilization of antibodies using amine coupling facilitated by EDC/NHS chemistry. (**D**) The label-free electrochemical immunobiosensor detects target interactions by monitoring changes in the interfacial electron-transfer kinetics of the redox probe [Fe(CN)_6_]^4−/3−^ during antibody–antigen binding events (reproduced with permission from [22]).

MXene-based electrochemical biosensors have demonstrated exceptional sensitivity and versatility in pathogen detection. These platforms leverage advanced nanostructures and aptamer integration, enabling precise identification of multiple infectious biomarkers. Liu et al. [22] and colleagues have addressed a critical gap in clinical diagnostics by developing an innovative multiplexed detection platform for infectious disease biomarkers. The development of a novel multiplexed screen-printed electrode (SPE) platform facilitated the simultaneous quantification of three critical infectious disease biomarkers: hepatitis B surface antigen (HBsAg), human immunodeficiency virus antibodies (anti-HIV), and Treponema pallidum antibodies (anti-TP). This innovative system employed a sophisticated electrode modification strategy incorporating in situ synthesized AuNPs integrated with MXene multilayer nanostructures, which yielded enhanced analytical sensitivity through optimized surface area utilization and electron transfer efficiency (Figure 6). The analytical performance of the AuNP and MXene-SPE platform demonstrated broad dynamic ranges for HBsAg (0.05–1000 ng/mL), anti-HIV (0.35–140 ng/mL), and anti-TP (0.25–100 ng/mL), with corresponding LODs of 0.01, 0.11, and 0.10 ng/mL, respectively. Validation studies conducted in standard serum matrices revealed outstanding precision, with relative standard deviations consistently below 5.0%, demonstrating robust analytical performance in complex biological environments. These attributes, coupled with the platform’s cost-effectiveness and rapid response capabilities, positioned this technology as a promising advancement in POC testing methodologies, potentially revolutionizing diagnostic protocols in resource-limited settings.

Yellow fever virus (YFV) is an acute infectious pathogen associated with significant morbidity and mortality during its toxic phase. Due to the absence of targeted therapeutic interventions, researchers have emphasized the importance of rapid detection and prophylactic measures. Kwon et al. [80] fabricated a high-sensitivity electrochemical biosensor incorporating a truncated DNA aptamer/MXene heterolayer configuration. The research team synthesized DNA aptamers via systematic evolution of ligands by exponential enrichment (SELEX), enabling specific recognition of YFV NS1 protein. MXene integration enhanced electrical conductivity and aptamer binding capacity through increased surface area. The implementation of aptamer truncation protocols optimized production economics. Biosensor performance underwent evaluation through CV and electrochemical impedance spectroscopy (EIS). The platform demonstrated detection limits of 2.757 pM and 2.366 pM for YFV in phosphate-buffered saline and 10% human serum, respectively, with confirmed specificity through selectivity analyses. The developed biosensor system presented potential applications in early YFV diagnosis, facilitating timely intervention and outbreak management strategies.

A novel electrochemical biosensor was developed for the detection of Norovirus (NoV), a significant foodborne pathogen that causes widespread gastroenteritis outbreaks. The sensing platform incorporated a unique sandwich-type configuration utilizing Au@BP@Ti_3_C_2_-MXene and magnetic Au@ZnFe_2_O_4_@COF nanocomposites as key components. Through extensive experimentation, Liu et al. [81] demonstrated that the biosensor exhibited a linear response to NoV concentrations within a range of 0.01–105 copies/mL, achieving a remarkable LOD of 0.003 copies/mL. This exceptional sensitivity was attributed to the synergistic effect between the specific molecular recognition capabilities of the affinity peptide–aptamer pair and the enhanced catalytic properties of the engineered nanomaterials. Validation studies successfully quantified NoV in simulated food matrices, while clinical stool samples were analyzed effectively without requiring complex sample preparation protocols. The innovative biosensor platform showed considerable promise for the sensitive detection of NoV across diverse sample types, including food products, clinical specimens, and environmental matrices, representing a significant advancement in foodborne pathogen diagnostics and food safety monitoring systems.

The global healthcare system has faced unprecedented challenges due to the significant health implications of SARS-CoV-2 infection. The research team developed an efficient biosensing methodology for viral detection utilizing amino-functionalized probe DNA that exhibits complementarity to the target viral sequence. Bharti et al. [82] successfully demonstrated the immobilization of NH_2_-pDNA onto Ti_3_C_2_T_x_-MXene nanosheet-modified SPCE. Their innovative detection platform achieved rapid results within 12 min and showed remarkable sensitivity across a broad linear detection range (0.1 pM to 1 µM), with a detection limit of 0.004 pM. The biosensor’s performance in spiked serum samples revealed comparable sensitivity, achieving a detection limit of 0.003 pM within a linear range spanning from 1 pM to 1 µM, suggesting potential clinical applicability. The successful validation in serum matrices indicated promising potential for real-world diagnostic applications.

**Figure 7:**
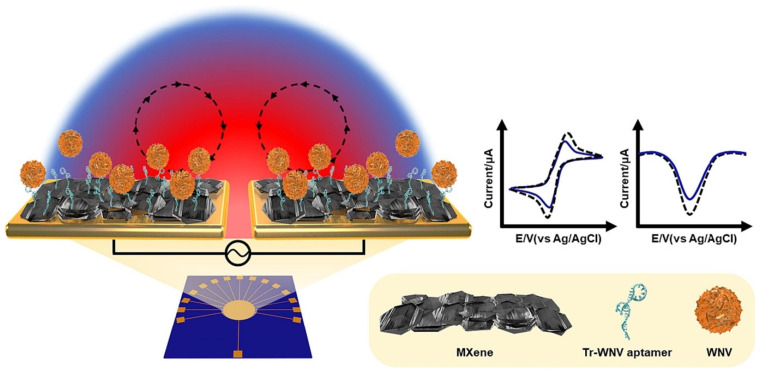
A schematic representation of the biosensor designed for rapid electrochemical detection of West Nile Virus (WNV), utilizing MXene/Tr-WNV aptamer and fabricated through ACEF technology (reproduced with permission from [83]).

Mosquito transmission of West Nile virus (WNV), a member of the flavivirus family, has been associated with severe neurological conditions including meningitis, encephalitis, and poliomyelitis, alongside West Nile fever. The necessity for field-based early detection systems highlighted the importance of developing cost-effective diagnostic tools. An investigation focused on engineering an expeditious electrochemical biosensing platform that incorporated a simplified WNV aptamer in conjunction with MXene (Ti_3_C_2_T_x_) in a bilateral configuration on a circular microinterval substrate. Park et al. [83] demonstrated that the integration of alternating current electrothermal flow (ACEF) methodology significantly enhanced the rapidity of target recognition, achieving detection within a 10 min timeframe (Figure 7). The implementation of MXene nanosheets facilitated enhanced electrochemical signal detection, while the systematic evolution of ligands by exponential enrichment generated an optimized aptamer through sequence truncation, thereby reducing production expenses. The analytical performance parameters, specifically the LOD and specificity, were evaluated utilizing electrochemical methodologies, encompassing CV and square wave voltammetric (SWV) techniques. The biosensor exhibited remarkable sensitivity, achieving LOD values of 2.57 pM and 1.06 pM for WNV in deionized water and 10% human serum matrices, respectively. The developed platform demonstrated exceptional selectivity coupled with rapid detection capabilities, suggesting its potential utility in Flaviviridae diagnostic applications.

### 4.3. Applications of MXene-Based Electrochemical Biosensors in Detecting Bodily Metabolites

**Figure 8:**
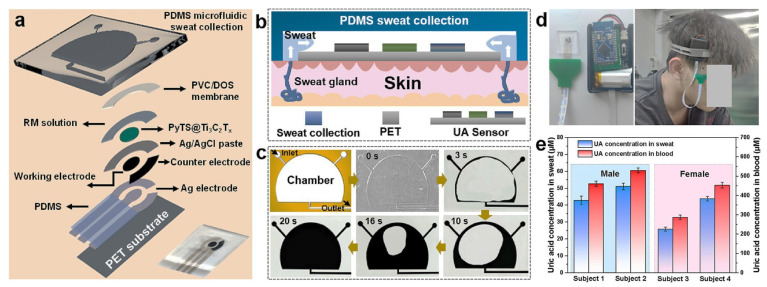
(**a**) Diagram showcasing the components of a flexible, wearable PyTS@Ti_3_C_2_T_x_-based microfluidic chip; (**b**) cross-sectional depiction of sweat collection through the chip; (**c**) schematic representation of the chip’s structure alongside images illustrating the sequential flow of black ink fluid through the inlet into the chamber over time; (**d**) photograph of a wearable device for on-body monitoring of sweat uric acid; and (**e**) correlation between on-body uric acid concentration measurements and blood uric acid levels (reproduced with permission from [21]).

The innovative application of MXene-based electrochemical biosensors demonstrated remarkable potential in the detection of various bodily metabolites. Contemporary healthcare monitoring has been revolutionized by developing continuous metabolite detection systems utilizing noninvasive wearable sensors, which facilitate the assessment of various health conditions through sweat analysis. The measurement of uric acid (UA) concentrations holds particular significance due to its established correlations with multiple pathological conditions, including gout, hyperuricaemia, hypertension, renal dysfunction, and Lesch–Nyhan syndrome; however, traditional diagnostic methods have predominantly relied upon invasive blood sampling techniques. The implementation of wearable technology for noninvasive UA monitoring in sweat presented a compelling alternative for real-time health surveillance. Chen et al. [21] addressed this challenge through the novel application of 1,3,6,8-pyrene tetrasulfonic acid sodium salt (PyTS), which was functionalized with Ti_3_C_2_T_x_ through π–π conjugation, resulting in the development of non-enzymatic wearable sensor systems capable of precise and specific UA detection in human sweat secretions (Figure 8). The integration of PyTS with Ti_3_C_2_T_x_ generated numerous oxidation–reduction active sites, thereby enhancing the electrocatalytic efficiency of UA oxidation processes. The electrochemical sensing platform incorporating PyTS@Ti_3_C_2_T_x_ demonstrated remarkable sensitivity within the UA concentration range of 5 μM–100 μM, achieving a detection threshold of 0.48 μM, which surpassed the capabilities of conventional uricase-based detection systems (0.84 μM). The practical application of this technology was validated through human trials, where the PyTS@Ti_3_C_2_T_x_-based wearable device, equipped with flexible microfluidic sweat collection mechanisms and wireless transmission capabilities, successfully monitored real-time UA concentrations during aerobic exercise, while simultaneously enabling comparative analysis with blood UA measurements obtained via commercial analytical instruments.

The detection of glucose is crucial for managing diabetes, as it enables timely monitoring and regulation of blood sugar levels, thereby preventing complications associated with the disease. Subramania et al. [84] successfully engineered a non-enzymatic biosensing platform through the strategic incorporation of NiCo_2_O_4_ nanoparticles into Ti_2_NbC_2_ nanosheets utilizing an in situ synthetic approach for the detection of glucose. The investigation of the nanohybrid’s electrocatalytic performance and electrochemical characteristics was accomplished through CV and amperometric analytical techniques. The resultant hybrid structure demonstrated biocompatibility and enhanced electrical conductivity with an electrochemically active interface. The amalgamation of NiCo_2_O_4_’s augmented surface area and the Ti_2_NbC_2_ nanosheet’s superior electrical conductivity facilitated the development of a non-enzymatic glucose detection system characterized by enhanced sensitivity (425.6 µA mM^−1^cm^−2^), a diminished detection threshold, and accelerated response kinetics, rendering it suitable for glucose monitoring applications.

The detection of lactate is crucial for assessing metabolic states in clinical settings, as it provides vital information regarding tissue hypoxia, exercise performance, and the prognosis of critically ill patients, enabling timely interventions in emergencies and optimizing athletic training regimens. The research endeavour of Liu et al. [85] was focused on the successful synthesis and integration of europium(III)-doped tin dioxide nanostructures within MXene (Ti_3_C_2_) laminar frameworks to generate Ti_3_C_2_@Eu-SnO_2_ hybrid materials towards lactate detection. They conducted comprehensive characterization studies employing CV measurements and EIS. Lactate oxidase (Lox)-functionalized Ti_3_C_2_@Eu-SnO_2_ demonstrated favourable hybrid coordination properties and significant biocompatibility among the Eu-SnO_2_, Ti_3_C_2_, and enzymatic components. The enzymatic electrochemical sensing platform, constructed through the immobilization of Ti_3_C_2_@Eu-SnO_2_/Lox onto a glassy carbon electrode (GCE) substrate, exhibited remarkable analytical performance with a linear response range spanning from 1.0 × 10^−9^ to 1.0 × 10^−4^ mol/L lactate concentrations. The biosensing system demonstrated exceptional analytical figures of merit, including a remarkably low detection threshold of 3.38 × 10^−10^ mol/L and a substantial sensitivity of 4.815 mA nmol^−1^ L cm^−2^. The practical applicability of the fabricated biosensor was validated through successful recovery experiments in human serum matrices.

### 4.4. Applications of MXene-Based Electrochemical Biosensors in Detecting Chemical Messengers

**Figure 9:**
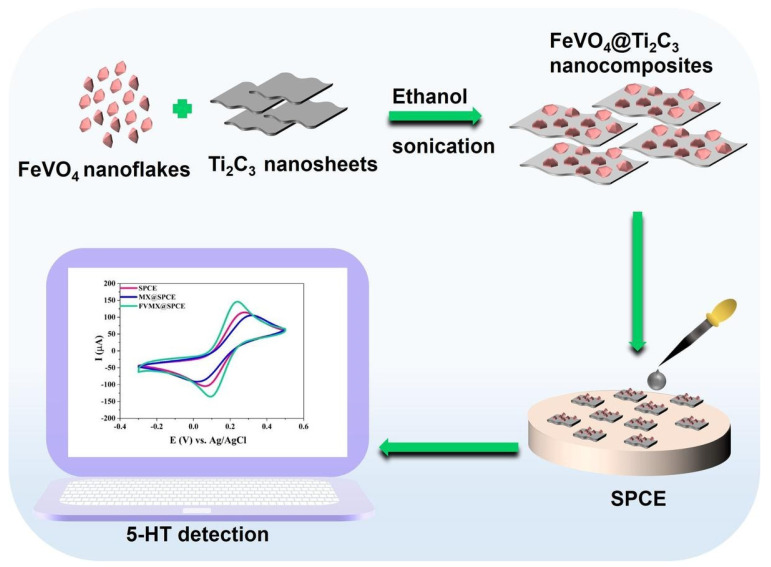
Schematic representation of the fabrication process of the FeVO_4_@Ti_3_C_2_ MXene-based biosensor and its application in 5-HT detection (reproduced with permission from [86]).

The detection of serotonin is crucial due to its significant role in regulating physiological processes and its association with various neurological and psychiatric disorders. Ilanchezhiyan et al. [86] investigated the fabrication of a novel electrochemical biosensor for serotonin (5-HT), a neurotransmitter critical to numerous physiological and pathological functions (Figure 9). The study emphasized the development of 2D FeVO_4_ nanoflakes (NFs) integrated with Ti_3_C_2_ MXene hybrid nanocomposites for the highly selective and ultrasensitive detection of 5-HT. The synthesis process was systematically characterized to confirm the morphology and crystalline phase of the nanocomposites, revealing uniform decoration of FeVO_4_ NFs on the Ti_3_C_2_ MXene surface. Electrochemical analysis using DPV and CV demonstrated significantly enhanced activity of the FeVO_4_@Ti_3_C_2_ MXene-modified SPCE for 5-HT oxidation. Under optimized conditions, the biosensor exhibited a linear detection range of 25 to 750 nM with a detection limit of 5.88 nM and a calculated sensitivity of 3.0 μA μM⁻^1^ cm⁻^2^. The fabricated biosensor further provided satisfactory performance in real-sample analysis, including human serum, showcasing its potential for pharmaceutical and diagnostic applications.

Dopamine detection is crucial for understanding its role in various neurological and psychiatric disorders, as it enables the assessment of dopaminergic signalling dynamics, which are essential for diagnosing conditions like Parkinson’s disease and schizophrenia, and for evaluating the efficacy of therapeutic interventions. Jing et al. [87] developed a ratiometric electrochemical biosensing platform utilizing Ti_3_C_2_T_x_ MXene for dopamine quantification. The research team employed an in situ reduction protocol to synthesize the MXene-Au hybrid material. They demonstrated the incorporation of methylene blue as an internal reference moiety onto the MXene-Au composite via electrostatically mediated interactions, resulting in the formation of MB-MXene-Au. The implementation of this reference molecule immobilization strategy demonstrated significant advantages, including procedural simplicity, enhanced stability characteristics, and reproducible performance metrics. The exceptional conductivity properties exhibited by both the MXene substrate and gold nanoparticles contributed to the biosensor’s remarkable sensitivity in dopamine detection. The analytical performance of the developed ratiometric sensing platform was validated through dopamine quantification in clinical human serum specimens, yielding an LOD of 0.04 μM and demonstrating linear response characteristics across a concentration range spanning from 0.1 μM to 100 μM, thus establishing its viability for practical applications.

### 4.5. Applications of MXene-Based Electrochemical Biosensors in Other Biomedical Applications

**Figure 10:**
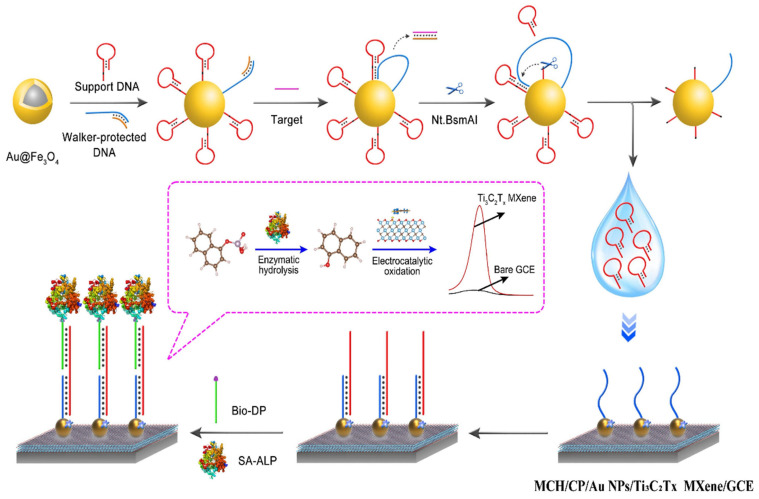
Schematic representation of the working principle of an electrochemical biosensor for detecting the BCR-ABL1 fusion gene (reproduced with permission from [88]).

The detection of leukemia through the BCR-ABL1 fusion gene is essential for early diagnosis, prognosis, and targeted therapy, particularly in chronic myeloid leukemia and Philadelphia chromosome-positive acute lymphoblastic leukemia. Early and accurate identification allowed timely intervention with tyrosine kinase inhibitors, improving patient outcomes and survival rates. Yu et al. [88] investigated the nanozyme-like behaviour of Ti_3_C_2_T_x_ MXene, which exhibited area-dependent electrocatalytic activity in phenolic compound oxidation. This behaviour was attributed to strong adsorption interactions between phenolic hydroxyl groups and oxygen-terminated surface sites on Ti_3_C_2_T_x_ MXene nanosheets. Based on this catalytic mechanism, the researchers combined Ti_3_C_2_T_x_ MXene with alkaline phosphatase to create a cascade amplification system using 1-naphthyl phosphate (1-NPP) as a substrate. This system enhanced electrochemical signal generation through sequential enzymatic and nanozyme-mediated catalytic conversion. Using this dual-catalytic approach, an electrochemical biosensor was developed for the ultrasensitive detection of the BCR-ABL1 fusion gene (Figure 10). The biosensor demonstrated a linear response ranging from 0.2 fM to 20 nM and achieved a detection limit of 0.05 fM. This platform provided a clinically relevant tool for the early diagnosis of chronic myeloid leukemia and acute lymphoblastic leukemia.

Bioelectronic tongues incorporating taste receptors have recently emerged as systems capable of mimicking human gustatory perception. However, receptor-based biosensors previously faced limitations due to their restricted dynamic ranges and low sensitivity. To address this, Liu et al. [89] introduced an innovative immobilization approach employing AuNPs@ZIF-8/Ti_3_C_2_ MXene nanocomposites to stabilize the umami-sensitive ligand-binding domain (T1R1-VFT), enabling the fabrication of a biosensor for umami compound detection. The synergistic combination of AuNPs@ZIF-8 and Ti_3_C_2_ MXene enhanced T1R1-VFT loading capacity and amplified electrochemical response signals by approximately threefold. The developed biosensor exhibited an ultrawide dynamic detection range from 10⁻^11^ to 10⁻^3^ M, with an upper detection limit closely aligned with human taste thresholds, making it suitable for analyzing umami-rich food products. They further validated the biosensor’s practicality by successfully detecting umami compounds in real samples and evaluating synergistic interactions between binary umami substances. This advancement highlights the potential of hybrid nanomaterials in overcoming historical limitations of bioelectronic taste-sensing platforms.

The prior discussion of electrochemical biosensors based on MXene for biomedical applications is summarized in Table 2.

## 5. Challenges and Future Perspectives

MXene-based electrochemical biosensors have demonstrated remarkable potential for biomedical applications, exhibiting high sensitivity, LODs, and rapid response times for a variety of biomarkers. As evidenced by Table 2, these biosensors have been successfully applied to detect various targets, including proteins (e.g., folate receptor, tPSA, fPSA, CEA, NS1 protein, WNV envelope protein), nucleic acids (e.g., miRNA-21, miRNA-122, microRNA-155, SARS-CoV-2), viruses (e.g., Norovirus), and small molecules (e.g., uric acid, glucose, lactate, serotonin, dopamine), in different biological samples such as serum, urine, sweat, and stool. The integration of MXenes with other nanomaterials, such as metal nanoparticles (AuNPs), metal oxides (NiCo_2_O_4_, Eu-SnO_2_, FeVO_4_), polymers (PEDOT:PSS, PAMAM, PnBA), and carbon nanomaterials (rGO, f-graphene), has further enhanced their performance.

MXenes, a class of two-dimensional transition metal carbides, nitrides, or carbonitrides, exhibit variable stability depending on their composition, surface terminations, and environmental conditions [90]. While inherently stable in inert or vacuum environments, MXenes are prone to oxidative degradation when exposed to oxygen, moisture, or elevated temperatures, particularly in aqueous media, which can lead to the formation of metal oxide layers and diminished electrical/mechanical performance [91]. The stability is influenced by surface functional groups (-O, -OH, -F) introduced during synthesis (e.g., HF etching), with oxygen-terminated MXenes generally showing higher resistance to oxidation compared to fluorine-rich surfaces [92]. Developing robust stabilization strategies, such as surface functionalization [93] or hybridization [94] with other materials, is essential to enhance their resilience in complex biological matrices.

Furthermore, the linear detection ranges must be tailored to accommodate varying clinical requirements, as seen in the broad detection range for Norovirus (0.01 to 10⁵ copies/mL) and more constrained ranges for serotonin (25 to 750 nM). Ensuring consistent performance across diverse analytes and sample types is critical for broad-spectrum applicability [95].

The biocompatibility and toxicity of MXenes are additional concerns [96], particularly for in vivo applications. MXenes, particularly Ti_3_C_2_-based variants, have been extensively studied for their biocompatibility and interactions with biological systems. In vitro experiments [50] reveal that MXenes generally exhibit low cytotoxicity at controlled concentrations (<100 µg/mL) across cell lines such as fibroblasts, epithelial cells, and macrophages. Their hydrophilic surfaces and tunable functional groups (-O, -OH) promote favourable interactions with cellular membranes, facilitating applications like drug delivery and bioimaging. However, dose-dependent cytotoxicity emerges at higher concentrations due to ROS generation or physical membrane disruption, particularly with smaller, unmodified MXene nanosheets [97]. In vivo studies in rodent models demonstrate that MXenes [98], when administered intravenously or subcutaneously, are largely biocompatible but exhibit context-dependent biodistribution and clearance. While acute inflammatory responses (e.g., transient neutrophil infiltration) are observed at high doses, chronic studies show minimal systemic toxicity, suggesting tolerable short-term biocompatibility [99]. Moreover, MXenes exhibit low inherent immunostimulatory effects under optimal conditions. In vitro assays with immune cells (e.g., macrophages, dendritic cells) show negligible pro-inflammatory cytokine release (e.g., TNF-α, IL-6) at biocompatible doses [100]. However, surface chemistry and functionalization critically influence the final outcome. Current evidence suggests that MXenes are not inherently immunogenic but require surface engineering and dose optimization to mitigate context-dependent immune activation [101]. Further long-term in vivo studies are essential to validate their safety for biomedical applications, such as implantable sensors or targeted therapies.

Integration of these biosensors into portable, user-friendly devices presents another challenge, particularly in maintaining sensitivity and specificity while miniaturizing the systems. The reported detection limits, while impressive (reaching as low as 0.0035 aM), need to be consistently achievable in complex biological matrices. The integration of wearable sensors, microfluidic systems, and artificial intelligence (AI)-based analysis enables continuous, real-time health monitoring with enhanced precision and automation. Wearable MXene-based biosensors [102] offer high sensitivity and flexibility, allowing noninvasive detection of biomarkers through sweat, saliva, or interstitial fluids. Microfluidic systems integrated with MXene [76] enhance sample handling, enabling precise fluid control and minimal reagent consumption, improving sensor efficiency and response time. AI-driven data analysis, incorporating machine learning algorithms, enables pattern recognition, anomaly detection, and predictive diagnostics, facilitating early disease detection and personalized healthcare [103]. The seamless combination of these technologies holds great potential for next-generation smart MXene-based biosensing platforms in remote and POC applications.

The standardization of fabrication processes and quality control measures are essential for commercial viability [104]. Moreover, the stability and reproducibility of these sensors are critical for their practical application. The environmental conditions under which these sensors operate can affect their performance, necessitating further research into protective coatings or hybrid materials that can enhance durability without compromising sensitivity.

The commercialization of MXene-based biosensors requires scalable synthesis, cost-effective fabrication, and regulatory approval. Optimizing manufacturing techniques, such as solution processing and inkjet printing, enhances production efficiency. Ensuring biocompatibility, stability, and accuracy through rigorous validation is essential for regulatory compliance. Industry collaborations and investment in pilot-scale production are crucial for transitioning these sensors from research to real-world applications in healthcare and environmental monitoring.

The disposal, degradation, and storage of MXene-based electrochemical sensors require careful consideration due to MXenes’ environmental sensitivity and potential ecological impact. MXenes, particularly those containing heavy metals (e.g., Ti_3_C_2_), may pose risks if improperly disposed of, necessitating protocols such as high-temperature incineration [105] or chemical neutralization [106] to minimize environmental release. Degradation of MXenes occurs primarily via oxidation in humid or aqueous environments, forming stable metal oxides (e.g., TiO_2_) and carbonaceous byproducts, though prolonged exposure to oxygen or moisture during storage can accelerate this process, compromising sensor performance. To mitigate degradation, MXene-based sensors are best stored in vacuum-sealed containers, while delaminated MXenes in colloidal suspensions require stabilization in organic solvents with airtight packaging. Recycling strategies, such as recovering MXene-derived oxides for secondary applications [107], are emerging to address end-of-life management. Proper handling ensures both sensor longevity and reduced ecological footprint, balancing their advanced functionality with sustainable lifecycle practices.

MXene-based electrochemical sensors offer potential for regeneration [108] through chemical treatments, surface modifications, or electrochemical cleaning to restore functionality. Strategies like mild oxidation, controlled voltage applications, and self-healing coatings can enhance reusability. However, the effectiveness depends on sensor composition and degradation mechanisms, requiring further optimization.

Future developments should focus on improving the long-term stability of MXene-based platforms, possibly through novel surface protection strategies or composite formations. Research efforts should target reducing response times while maintaining high sensitivity, particularly for POC applications. The development of multiplexed sensing platforms capable of simultaneous detection of multiple biomarkers would significantly enhance diagnostic capabilities. Integration with emerging technologies such as artificial intelligence (AI) for data analysis and wireless communication for remote monitoring could expand the application scope of these biosensors in personalized medicine and continuous health monitoring. Furthermore, the integration of MXenes into wearable technology holds great promise for real-time health monitoring and POC diagnostics, particularly in tracking chronic diseases. Collaborative efforts between material scientists, biochemists, and clinicians will be crucial in addressing these challenges, paving the way for MXene-based biosensors to become indispensable tools in biomedical diagnostics and therapeutic monitoring.

## 6. Conclusions

This comprehensive review depicted that MXene-based electrochemical biosensors represent a significant advancement in biomedical detection technologies, showcasing exceptional versatility and performance across diverse biomedical applications. The integration of MXenes with various nanomaterials has enabled the development of highly sensitive biosensing platforms capable of detecting a wide range of biomarkers, from proteins and nucleic acids to viruses and small molecules. These biosensors have demonstrated practical utility across different biological matrices, including serum, urine, sweat, and stool samples, while maintaining impressive sensitivity and specificity. The successful detection of disease-specific biomarkers for conditions ranging from cancer and infectious diseases to metabolic disorders highlights the broad potential of MXene-based biosensors in clinical diagnostics. While challenges persist regarding material stability, standardization, and system integration, ongoing research advances in surface modification strategies, composite materials, and device fabrication continue to address these limitations. The integration of MXene-based biosensors with emerging technologies such as AI and wireless communication, coupled with their potential in wearable devices, positions them as promising candidates for next-generation biomedical diagnostics and personalized healthcare monitoring systems.

## Figures and Tables

**Table 1:** General comparison of MXene with other 2D nanomaterials (graphene, transition metal dichalcogenides (TMDs), and hexagonal boron nitride (h-BN)) in terms of composition, properties, and applications in electrochemical devices.

Property	MXene	Graphene	Transition Metal Dichalcogenides (TMDs)	Hexagonal Boron Nitride (h-BN)
Composition	Transition metal carbides/nitrides with surface functional groups (e.g., -O, -OH, -F)	Carbon atoms in a hexagonal lattice	Transition metal (e.g., Mo, W) sandwiched between chalcogen atoms (e.g., S, Se)	Boron and nitrogen atoms in a hexagonal lattice
Electrical Conductivity	High electrical conductivity	Extremely high electrical conductivity	Semiconducting (varies with material and structure)	Insulating
Mechanical Strength	High mechanical strength and flexibility	Extremely high mechanical strength	Moderate mechanical strength	High mechanical strength
Surface Chemistry	Tunable surface functional groups, hydrophilic nature	Inert surface, hydrophobic unless functionalized	Limited surface reactivity, can be functionalized	Chemically inert, hydrophobic
Bandgap	Metallic or semiconducting (tunable based on composition and functionalization)	Zero bandgap (semimetal)	Tunable bandgap (direct/indirect depending on layers)	Wide bandgap (insulator)
Processability	Easily dispersible in water and other solvents, good for solution processing	Requires functionalization for dispersion, prone to aggregation	Moderate dispersibility, often requires exfoliation	Poor dispersibility, requires exfoliation or functionalization
Catalytic Activity	High catalytic activity for electrochemical reactions	Moderate catalytic activity, can be enhanced with doping/defects	Good catalytic activity	Low catalytic activity
Stability	Moderate stability in air, can oxidize over time	High stability in inert environments, can oxidize in air	Moderate to high stability depending on material	Excellent thermal and chemical stability
Applications	Supercapacitors, batteries, sensors, electrocatalysis, EMI shielding	Supercapacitors, batteries, sensors, transparent conductors	Transistors, photodetectors, HER catalysts, batteries	Insulators, substrates, thermal management, protective coatings

**Table 2:** Key parameters of MXene-based electrochemical biosensors for biomedical applications.

Material Composition	Target	Sample Type	LOD	Sensitivity	Linear Range	Response Time	Disease	Ref.
PAMAM@ Ti_3_C_2_T_X_	Folate Receptor	NA	5.6 ng/mL	NA	10 ng/mL to 1000 ng/mL	40 min	Tumour	[79]
Ti_3_C_2_@PEDOT: PSS	tPSA and fPSA	NA	5.3 fg/mL for fPSA; 4.8 fg/mL for tPSA	NA	0.01 pg/mL to 100 ng/mL	40 min	Prostate cancer	[46]
f-graphene@Ti_3_C_2_	CEA	NA	0.30 pg/mL	28.88 μA [log (pg mL^−1^)]^−1^ cm^−2^	0.01 pg/mL to 2000 ng/mL	10 min	Cancer	[71]
Ti_3_C_2-_rGO-Au	miRNA-21	Urine	0.42 fM	NA	1 fM to 1 nM	2 h	Cancer	[20]
Au HFGNs/PnBA-MXene	miRNA-122	Serum	0.0035 aM	NA	0.01 aM to 10 nM	30 min	Breast Cancer	[23]
AuNPs/Ti_3_C_2_	microRNA-155	NA	0.35 fM	NA	1.0 fM to 10 nM	2 h	Cancer	[72]
AuNP/Ti_3_C_2_T_x_	HBsAg, anti-HIV, and anti-TP.	Serum	0.01 (HBsAg), 0.11 (anti-HIV), and 0.10 ng/mL (anti-TP)	NA	0.05 to 1000 ng/mL (HBsAg), 0.35 to 140 ng/mL (anti-HIV), and 0.25 to 100 ng/mL (anti-TP)	45 min	Hepatitis B, Syphilis, and AIDS	[22]
Truncated DNA aptamer/MXene	NS1 protein	Serum	2.366 pM	NA	10 pM to 1 μM	3 h	Yellow fever	[80]
Au@BP@Ti_3_C_2_	Norovirus	Stool	0.003 copies/mL	NA	0.01 to 10^5^ copies/mL	2 h	Acute gastroenteritis	[81]
NH_2_-pDNA/Ti_3_C_2_T_x_	SARS-CoV-2	Serum	0.003 pM	NA	1 pM to 1 µM	12 min	COVID-19	[82]
Truncated WNV aptamer/Ti_3_C_2_T_x_	WNV envelope protein	Serum	1.06 pM	NA	10 pM to 1 μM	10 min	West Nile fever, meningitis, and polio	[83]
PyTS@Ti_3_C_2_T_x_	Uric Acid	Sweat	0.48 μM	NA	5 μM to 100 μM	20 min	Gout, kidney disease	[21]
NiCo_2_O_4_/Ti_2_NbC_2_	Glucose	Sweat	0.298 mM	425.6 µA mM^−1^cm^−2^	0.02 to 1 mM	2 min	Diabetes	[84]
Ti_3_C_2_@Eu-SnO_2_	Lactate	Serum	3.38 × 10^−10^ mol/L	4.815 mA nmol^−1^ L cm^−2^	1.0 × 10^−9^ to 1.0 × 10^−4^ mol/L	NA	Tissue hypoxia	[85]
FeVO_4_/Ti_3_C_2_	Serotonin	Serum	5.88 nM	3 μA/µΜ	25 to 750 nM	NA	Depression and cardiovascular disorders	[86]
MB/Ti_3_C_2_T_x_/Au	Dopamine	Serum	0.04 μM	NA	0.1 μM to 100 μM	NA	Neurological disorder	[87]
MCH/CP/AuNPs/Ti_3_C_2_T_x_ MXene	BCR/ABL fusion gene	Serum	0.05 fM	NA	0.2 fM to 20 nM	1 h	Leukemia	[88]
T1R1-VFT/AuNPs@ZIF-8/Ti_3_C_2_	Umami substances (MSG, IMP, WSA, BMP)	Soy sauce, tomato juice, green tea and cola	10.0 pM (MSG), 10.0 pM (IMP), 10.0 pM (WSA), 1.0 × 10^3^ pM (BMP)	NA	1.0 × 10^−2^ to 1.0 × 10^6^ nM (MSG) 1.0 × 10^−2^ to 1.0 × 10^3^ nM (IMP), 1.0 × 10^−2^ to 1.0 × 10^5^ nM (WSA) and 1.0 to 1.0 × 10^5^ nM (BMP)	20 min	Taste disorders	[89]
